# Oxide Materials for Thermoelectric Conversion

**DOI:** 10.3390/molecules28155894

**Published:** 2023-08-05

**Authors:** Yucen Liu, Jun Zhi, Wannuo Li, Qian Yang, Long Zhang, Yuqiao Zhang

**Affiliations:** 1 Institute of Quantum and Sustainable Technology (IQST), School of Chemistry and Chemical Engineering, Jiangsu University, Zhenjiang 212013, China; yc_liu1999@163.com (Y.L.); 2222212112@stmail.ujs.edu.cn (J.Z.); lwn000119@163.com (W.L.); 2 Foshan (Southern China) Institute for New Materials, Foshan 528200, China

**Keywords:** thermoelectrics, oxide, ZnO, SrTiO_3_, layered cobalt oxides

## Abstract

Thermoelectric technology has emerged as a prominent area of research in the past few decades for harnessing waste heat and improving the efficiency of next-generation renewable energy technologies. There has been rapid progress in the development of high-performance thermoelectric materials, as measured by the dimensionless figure of merit (*ZT* = *S*^2^ · *σ* · *κ*^−1^). Several heavy-metal-based thermoelectric materials with commercial-level performance (*ZT* = 1) have so far been proposed. However, the extensive application of these materials still faces challenges due to their low thermal/chemical stability, high toxicity, and limited abundance in the Earth’s crust. In contrast, oxide-based thermoelectric materials, such as ZnO, SrTiO_3_, layered cobalt oxides, etc., have attracted growing interest as they can overcome the limitations of their heavy-metal-based counterparts. In this review, we summarize the recent research progress and introduce improvement strategies in oxide-based thermoelectric materials. This will provide an overview of their development history and design schemes, ultimately aiding in enhancing the overall performance of oxide-based thermoelectric materials.

## 1. Introduction

Following the progression of human civilization and industrialization, vast amounts of fossil fuels have been consumed for energy purposes [1]. However, due to the low efficiency of utilizing fossil fuels, only around 30% of the primary energy can be effectively harnessed, while more than 60% is wasted as heat [2]. Therefore, the exploration of new and efficient technologies for recycling waste heat is crucial to align with the sustainable world envisioned by the United Nations in 2015 [3]. 

**Figure 1:**
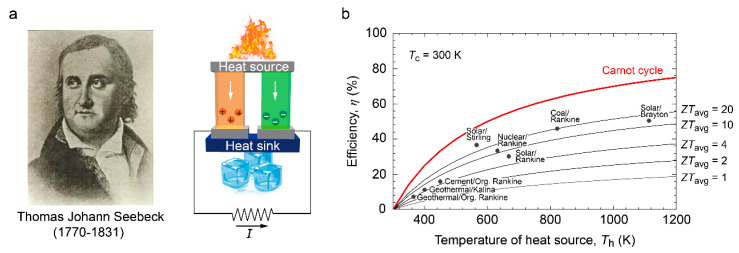
Schematic illustration of thermoelectric generators. (**a**) Thermoelectric power generation process based on the Seebeck effect, which was discovered by Thomas Johann Seebeck (1770–1831). Using a temperature gradient applied along the thermoelectric P-N junction, charge carriers inside the materials are driven from the hot side to the cold side, resulting in a current flow (*I*) through the circuit. (**b**) Power generation efficiency (*η*), as a function of the temperature of heat source (*T*_h_), in the thermoelectric generators with different average *ZT* values (*ZT*_avg_). The efficiency values for several heat engines currently being used are plotted for comparison. Reproduced with permission [7].

Thermoelectrics has emerged as a promising technique for the reutilization of waste heat [4,5]. By leveraging the Seebeck effect, thermoelectric materials can directly convert a temperature gradient into electricity (Figure 1a) [6]. The performance of a thermoelectric material is commonly quantified by a dimensionless figure of merit known as *ZT*, which is defined as:

$$ZT\;=\frac{S^{2}\sigma T}{\kappa }$$

where *S*, *σ*, *κ*, and *T* denote the Seebeck coefficient (or thermopower), electrical conductivity, thermal conductivity, and absolute temperature, respectively. The overall conversion efficiency (*η*) of a thermoelectric generator can be determined by *ZT* using the following relationship [1]:

$$\eta =\frac{\Delta T}{T_{h}}\;\cdot \;\frac{\sqrt{1+ZT}-\;1}{\sqrt{1+ZT}+\frac{T_{c}}{T_{h}}}$$

where *T*_h_, *T*_c_, and Δ*T* are the temperature at the hot and cold sides, and the temperature gradient along the thermoelectric generator, respectively. In accordance with the second law of thermodynamics, the maximum efficiency of the thermoelectric generators is constrained by the Carnot efficiency (Δ*T*/*T*_h_). As illustrated in Figure 1b, the conversion efficiency of the thermoelectric generators, with varying average *ZT* values (*ZT*_avg_), is plotted against *T*_h_ while assuming a fixed *T*_c_ at room temperature (300 K). To maximize *η*, it is imperative to utilize thermoelectric materials with high *ZT* values. Typically, *ZT*~1 is considered the minimum threshold for the practical application of a thermoelectric material.

Thus far, a range of thermoelectric materials exhibiting high *ZT* values has been discovered, including filled skutterudites alloy [8], semi-Heusler intermetallic compounds [9], Bi_2_Te_3_ [10], Mg_3_Sb_2_ [11], GeTe [12], AgPb*_m_*SbTe_2+*m*_ [13], Cu_2_Se [14], Cu_2_S [15], SnSe [16,17,18,19,20], etc, primarily belonging to the chalcogenide family. However, despite advancements, the conversion efficiency of thermoelectric materials still falls short of meeting practical application requirements when compared to traditional energy conversion techniques [7]. Additionally, these materials face limitations in high-temperature usage (i.e., >700 °C) due to their low thermal and chemical stabilities. Furthermore, their low natural element abundance and high toxicity introduce uncertainties and challenges in their practical implementation. 

To address these challenges, considerable attention has been directed toward oxide-based thermoelectrics as a potential solution. Oxide-based thermoelectric materials offer higher thermal and chemical robustness, making them more suitable for high-temperature applications. Moreover, as depicted in Figure 1b, the advantage of operating at high temperatures can offset some of the limitations associated with lower *ZT* values.

Extensive research on oxide-based thermoelectrics initially centered around investigating simple conducting oxides, such as CdO [21], NiO [22], ZnO [23], In_2_O_3_ [24], rutile-TiO_2_ [25], SnO_2_ [26], Cu_2_O [27], and Fe_3_O_4_ [28]. This was followed by a subsequent surge of interest in cuprous oxide-based superconducting materials, such as La_2_CuO_4_ [29], La-Ba-Cu-O [30], YBa_2_Cu_3_O_7−*δ*_ [31], and Tl-Ca-Ba-Cu-O [32]. Recent studies have uncovered the potential of compounds, including CaMnO_3_ [33], Zn_1−*x*_Al*_x_*O [34], *A_x_*CoO_2_ (*A_x_* = Na_0.75_, Ca_0.33_, Sr_0.33_, Ba_0.33_) [35,36,37,38,39,40,41], and SrTiO_3_ [42,43,44,45,46]. This review provides an overview of the advancements in oxide-based thermoelectrics, with a particular focus on the development of several representative compounds and the recent effective methods employed by researchers to enhance their properties. Notably, the review delves into the progress made in ZnO, SrTiO_3_, and layered cobaltates (*A_x_*CoO_2_, *A_x_* = Na_0.75_, Ca_0.33_, Sr_0.33_, Ba_0.33_). 

## 2. ZnO

**Figure 2:**
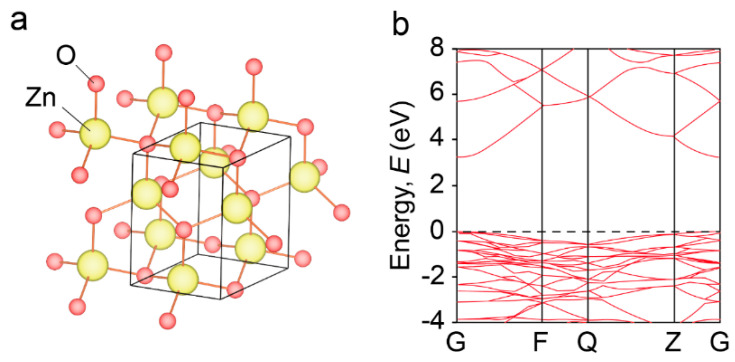
Schematic illustrations of (**a**) crystal structure and (**b**) electronic band structure of ZnO bulk materials. The pure ZnO exerts a wurtzite structure with a direct band gap of 3.3–3.5 eV [52].

ZnO is one of the most investigated oxides for photovoltaic, sensing, piezoelectric, and thermoelectric applications, with n-type semiconducting behaviors [34,47,48,49,50]. As shown in Figure 2a, ZnO crystals possess a wurtzite structure, where the Zn atom is surrounded by four oxygens, resulting in a hexagonal closely packed sublattice. In the solid state, ZnO acts as an insulator with a wide bandgap of about 3.2–3.5 eV. The conduction band minimum (CBM) is mainly derived from the 4s orbital in the Zn^2+^ ion, which shows a high carrier mobility. Pristine ZnO displays an increasing electrical conductivity with temperature due to the thermally excited electrons. Under ideal stoichiometric conditions, ZnO materials demonstrate a thermoelectric power factor ranging from 0.8 to 1 mW m^−1^ K^−2^. However, due to the low electrical conductivity and high thermal conductivity (49 W m^−1^ K^−1^ at 300 K and 10 W m^−1^ K^−1^ at 1000 K [51]), the *ZT* value and overall efficiency of pristine ZnO are still limited in practical applications. 

**Figure 3:**
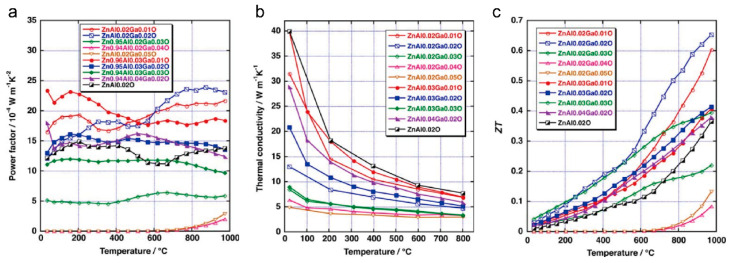
Thermoelectric properties of Al–Ga-co-doped ZnO. (**a**) Power factor. (**b**) Thermal conductivity. (**c**) *ZT* values. Reproduced with permission [57].

In order to enhance the electrical characteristics of zinc oxide (ZnO), the introduction of donor dopants such as Al^3+^ and Ga^3+^ has been widely employed. A small number of dopants can tune the conduction behavior of ZnO from semiconducting to metallic. In 1996, Ohtaki and Tswbota reported on the high-temperature thermoelectric properties of Al-doped ZnO, exhibiting a thermoelectric figure of merit (*ZT*) of approximately 0.3 at 1273 K, along with a thermoelectric power factor ranging from 1 to 1.5 mW m^−1^ K^−2^, which is comparable to traditional chalcogenide-based counterparts [34,50]. In 2011, Jood et al. prepared ZnO nanocomposites incorporating Al, further enhancing the *ZT* value to 0.44 at 1000 K [53]. In addition to Al, a diverse range of dopants, including Ga, Ni, Co, Fe, In, Ti, Mn, and Sb, have been utilized to improve either the overall or specific ZnO performance characteristics [54,55,56]. Furthermore, apart from their influence on electrical properties, elemental doping has proven effective in reducing the thermal conductivity in the ZnO system. As shown in Figure 3, Ohtaki et al. found that using a third element, Ga, co-doped with Al, could dramatically reduce the thermal conductivity, while having a relatively small effect on electrical conductivity [57]. With an obvious reduction in thermal conductivity and enhancement in power factor, the *ZT* value of the double-doped oxide in the composition of Zn_0.96_Al_0.02_Ga_0.02_O is 0.47 at 1000 K and 0.65 at 1247 K. 

**Figure 4:**
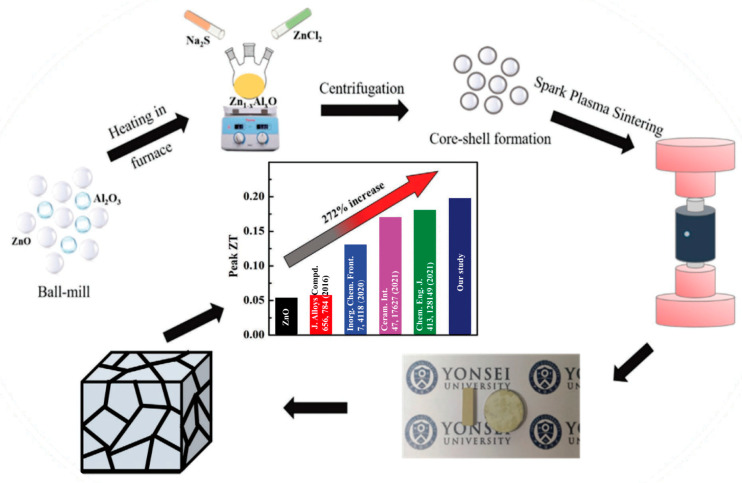
Schematic illustrating the synthesizing steps of ZnO-coated Zn_1−*x*_Al*_x_*O samples demonstrating the high *ZT* values compared to previously reported Al-doped ZnO. Reproduced with permission [58].

In 2021, utilizing the thermal conductivity reduction effect of the coating grain structure, Somnath Acharya et al. prepared ZnS-coated Al-doped compact ZnO nanostructures using the low-temperature co-precipitation method and spark plasma sintering (SPS), in which the Al doping increased the carrier concentration as well as optimizing the electron transport characteristics (Figure 4). The decrease in thermal conductivity can be attributed to the enhanced coherent phonon-scattering in Zn_1−*x*_Al*_x_*O. The power factor of the resulting 2% ZnS-coated Zn_0.98_Al_0.02_O nanostructures exhibited a noteworthy enhancement of 161% compared to pure ZnO, reaching a value of 0.75 mW m⁻^1^ K⁻^2^. Furthermore, a peak figure of merit (*ZT*) value of 0.2 was attained, which represents a remarkable 272% increase compared to pure ZnO [58].

**Figure 5:**
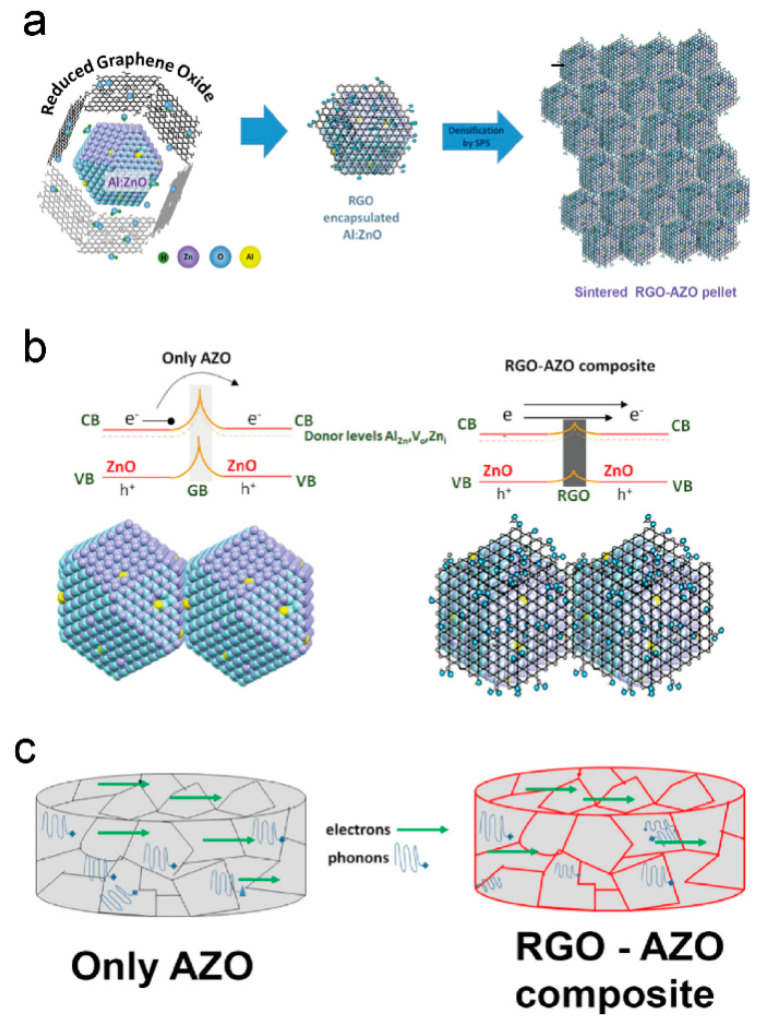
Schematic diagram of (**a**) formation of the RGO-encapsulated Al-doped ZnO composites (RGO–AZO). (**b**) Scattering and transport mechanisms in a densified ZnO–RGO composite across grain boundaries. (**c**) Scattering of phonons and electrons in bulk samples. Reproduced with permission [59].

More recently, as shown in Figure 5, Soumya Biswas et al. prepared ZnO nanocomposites using a simple solution-based method with both Al doping and reduced graphene oxide (RGO) encapsulation techniques. The influence of Al doping on the nanocomposites resulted in an increased electrical conductivity and decreased thermal conductivity, albeit accompanied by low Seebeck coefficient values. Furthermore, the incorporation of RGO inclusions into the nanocomposites yielded dual benefits. Firstly, it enhanced the overall electrical conductivity, contributing to improved charge transport properties. Secondly, through the phenomenon of energy filtration, the presence of RGO enhanced the Seebeck coefficient, promoting more favorable thermoelectric properties. Consequently, the synthesized material exhibited a notable figure of merit (*ZT*) value of approximately 0.52 at an elevated temperature of 1100 K [59].

**Figure 6:**
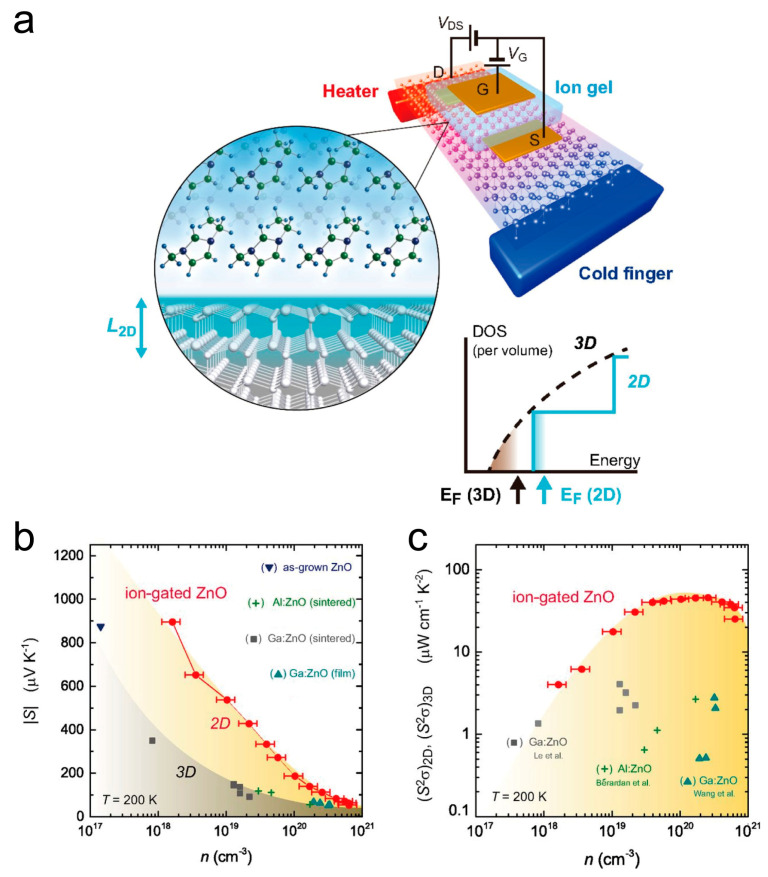
Two-dimensional electron gas accumulated at the surface of ZnO boosts the thermoelectric performance. (**a**) Schematic structure of ZnO-based ion-gated transistor. (**b**) Carrier concentration (*n*) dependence on Seebeck coefficient (*S*) at 200 K. The values of |*S*| in the ion-gated ZnO were larger than in bulk ZnO [62,63,64,65]. (**c**) Carrier concentration dependence of thermoelectric power factor (*S^2^σ*). The ion-gated ZnO showed larger values than the bulk ZnO, as expected from the enhanced *S*. Reproduced with permission [61].

In addition to the bulk state, recent progress has also been achieved in two-dimensional thin film-based ZnO materials. Zhou et al. prepared Ga-doped ZnO epitaxial thin films using pulsed laser deposition (PLD) technology. During the preparation process, growth rate, crystallinity, surface roughness, and even the Fermi level could be changed by controlling deposition temperature. The improved Ga doping efficiency resulted in an increased Hall mobility and higher carrier concentration, consequently, leading to a notable enhancement in electrical conductivity. Finally, GZO films deposited at 673 K showed the best quality and crystallinity, with a power factor of 256 mW m^−1^ K^−2^ [60]. In 2016, Shimizu et al. proposed that the thermoelectric performance of ZnO could be enhanced by a two-dimensional electron gas in the ion gel-gated field-effect transistor (Figure 6a) [61]. The accumulation of an ~1 nm 2D electron gas at the surface of ZnO can significantly enhance the Seebeck coefficient (Figure 6b), which brings about an obviously increased power factor compared to the bulk counterparts [62,63,64,65].

## 3. SrTiO_3_

**Figure 7:**
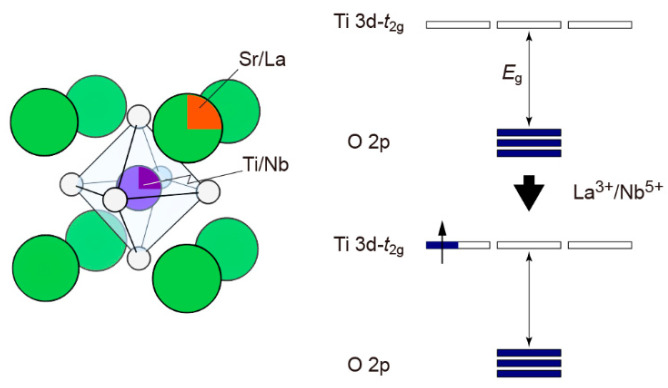
Schematic illustration of the SrTiO_3_ lattice structure and doping mechanism.

SrTiO_3_ is considered a promising n-type oxide thermoelectric material with a high Seebeck coefficient due to the large effective mass of the Ti 3D orbital [43]. Meanwhile, its high melting point also makes it a thermoelectric candidate at high temperatures. As shown in Figure 7, SrTiO_3_ has a simple cubic-perovskite crystal structure (lattice parameter, a = 3.905 Å). SrTiO_3_ behaves as an insulator, characterized by a very low carrier concentration, poor electron transport, and high thermal conductivity. Therefore, to enhance the thermoelectric performance of SrTiO_3_, two crucial aspects necessitate attention: optimizing the carrier concentration and reducing thermal conductivity. SrTiO_3_ possesses a high relative dielectric constant (*ε*_r_~220 at room temperature), and the impurity orbitals start to overlap at a rather low donor doping level (~0.5 mol%), resulting in a metallic electron transport property in SrTiO_3_ [43]. In this case, it is easy to introduce dopants (i.e., La^3+^ and Nb^5+^) into the SrTiO_3_ matrix and modulate the thermoelectric properties. 

**Figure 8:**
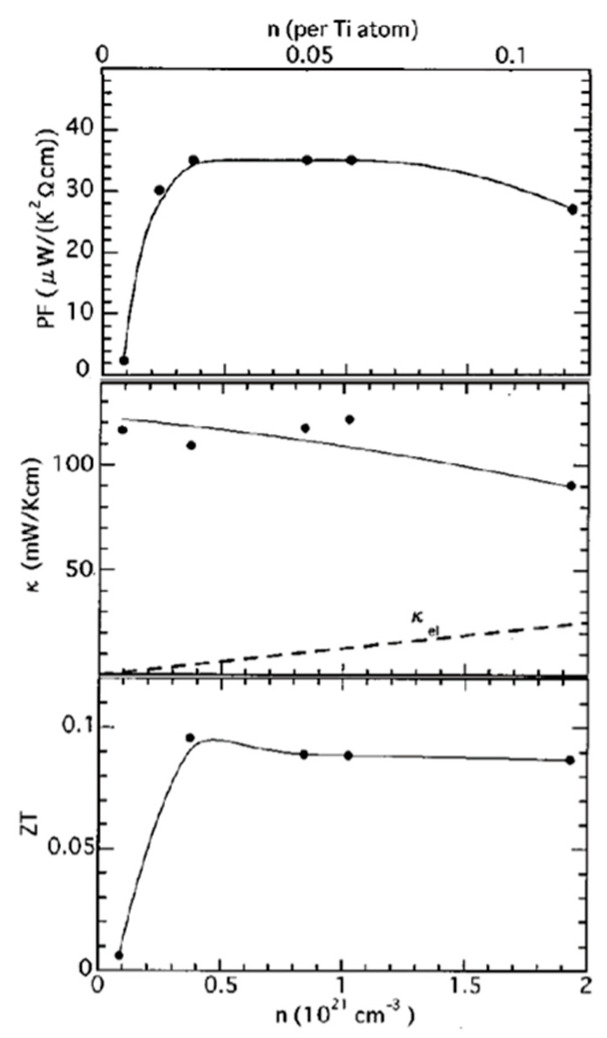
Carrier concentration (*n*) dependence of power factor (PF) thermal conductivity (*κ*), and *ZT* value at room temperature in Sr_1−*x*_La*_x_*TiO_3_. Solid lines are guides to the eye and the dashed line represents the electronic thermal conductivity (*κ*_el_), estimated by the Wiedemann–Franz law. Reproduced with permission [42].

In addition to the common donor dopant La^3+^ and Nb^5+^, other elements are also utilized to tailor the carrier concentration, lattice constant, defect level, and lattice distortion. In the A-site doping, common elements included La, Y, Na, Ce, Mn, Bi, Nd, Pr, Sm, Gd, Dy, etc. [5,66]. At the B-site, common doping elements included Nb, Ta, Se, V, Cr, Fe, Co, Ni, In, Sb, Mg, etc. Ru, Al, etc. [67]. In 2001, Okuda first found that the power factor of Sr_1−*x*_La*_x_*TiO_3_ was 28–36 mW cm^−1^k^−2^, which is comparable to traditional alloy-type thermoelectric material Bi_2_Te_3_ at room temperature, although its *ZT* was still < 1 due to its excessive thermal conductivity (Figure 8). Despite this limitation, the notable power factor exhibited by Sr_1−*x*_La*_x_*TiO_3_ proved its potential for application in thermoelectric systems [42].

**Figure 9:**
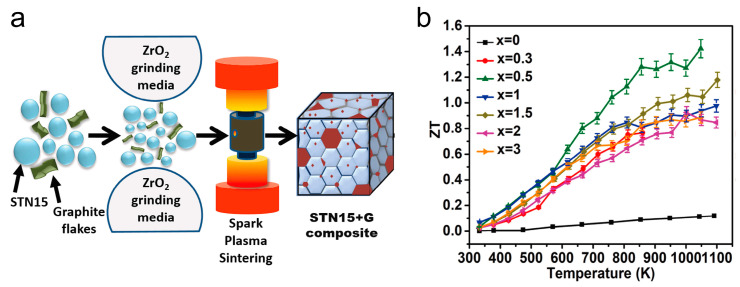
Schematic representation of processing bulk SrTi_0.85_Nb_0.15_O_3_ matrix (STN) oxide composites with graphite inclusions (**a**) and temperature-dependent *ZT* values for sintered STN + *x* wt% graphite composites (0 ≤ *x* ≤ 3) (**b**). Reproduced with permission [68].

Co-doping on both the Sr site and Ti site is a widely employed strategy in the field. In 2017, Wang et al. successfully overcame the challenges associated with nanoscale co-doping of Nb and La by integrating the hydrothermal method with efficient sintering techniques. Using this approach, they achieved precise control over the co-doping process, resulting in the precipitation of nano-inclusions during the sintering phase and the formation of a complex microstructure. Consequently, the Seebeck coefficient was significantly enhanced, while the thermal conductivity was effectively reduced. Finally, a record-high *ZT* of >0.6 was generated at 1000–1100 K in the 10 mol% La and 10 mol% Nb-doped SrTiO_3_ bulk materials [46]. More recently, Acharya et al. proposed thermoelectric composites of Nb-doped SrTiO_3_ using natural graphite, which possessed surged electrical conductivity, while restraining thermal conductivity (Figure 9). The *ZT* value can reach 1.42 at ~1050 K and keep within >1.2 of a broad temperature range (850–1050 K) [68]. 

**Figure 10:**
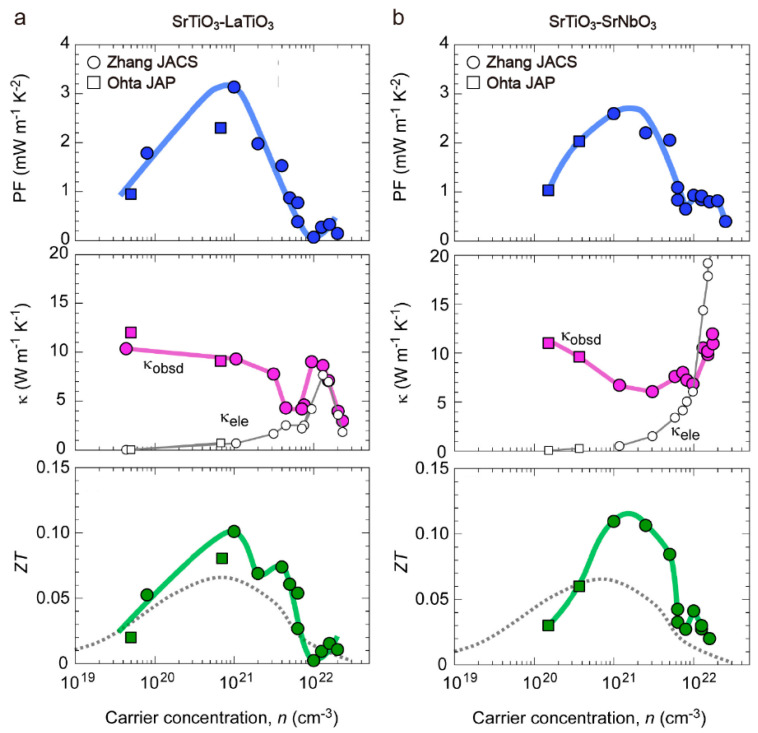
Power factor (PF), thermal conductivity (*κ*), and *ZT* values for SrTiO_3_–LaTiO_3_. (**a**) and SrTiO_3_–SrNbO_3_ (**b**) solid solutions. La^3+^ and Nb^3+^ substitutions result in a similar power factor behavior and a rather different tendency in thermal conductivity due to the different lattice distortions. The *ZT* value of SrTiO_3_-based epitaxial films reached 0.11 at room temperature. The solid line is a guide for eyes. The dotted lines are the values of bulk single crystals. Reproduced with permission [43,71].

In addition to common doping, low-dimensional is another effective strategy to improve thermoelectric performance. In thin film cases, phonon scatterings at the interface and surface areas will be much more obvious than in the bulk state. In 2021, Zhang et al. prepared La- and Nb-doped SrTiO_3_ full-range solid solution epitaxial films by the PLD method and investigated their thermoelectric phase diagrams at room temperature (Figure 10) [69,70]. Both SrTiO_3_–LaTiO_3_ and SrTiO_3_–SrNbO_3_ solid solutions displayed a similar power factor (=*S*^2^ · *σ*) changing pattern, which peaked at the carrier concentration (*n*) of around 10^21^ cm^−3^, while different thermal conductivity variation tendencies, especially at the substitution level, were above 50 mol%. The authors attributed this to the different lattice distortions in the two solid solution systems. Modulation of the thermal conductivity through doping achieved a much larger *ZT* value of ~0.11 at room temperature compared to previous reports [71]. 

**Figure 11:**
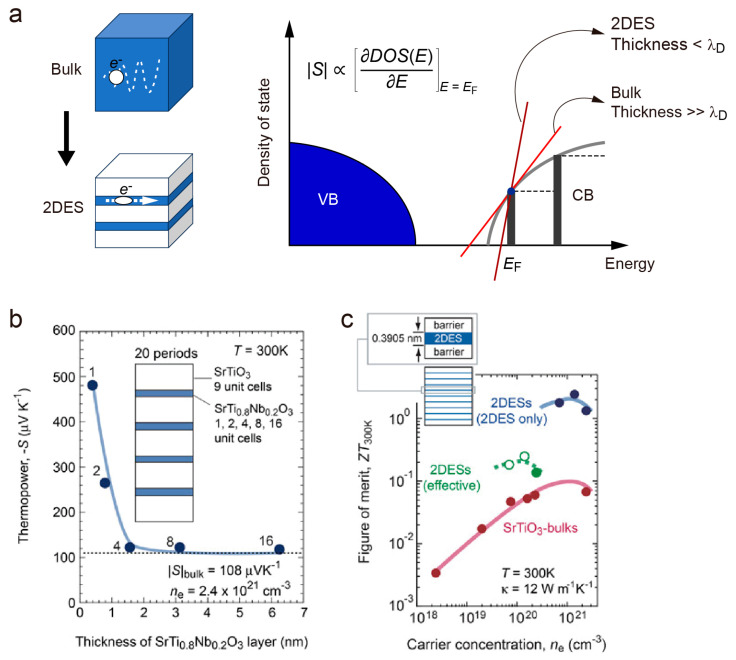
Enhancing thermopower through the two-dimensional electron system (2DES). (**a**) Schematic illustration of the 2DES enhanced thermopower mechanism while holding a good transport property. (**b**) Room temperature thermopower versus quantum well thickness in the SrTi_0.8_Nb_0.2_O_3_|SrTiO_3_ superlattice. An obvious boost in thermopower can be observed when the thickness of the SrTi_0.8_Nb_0.2_O_3_ quantum well is smaller than 1.56 nm (four-unit cells of SrTiO_3_). (**c**) Carrier concentration-dependent *ZT* values at room temperature. The *ZT* value of the SrTi_0.8_Nb_0.2_O_3_ quantum well reaches ~2.4. Reproduced with permission [73].

In 1993, Dresselhaus et al. published their findings that the two-dimensional electron system (2DES) can enhance thermopower without sacrificing electrical conductivity, which significantly boosted the *ZT* value [72]. As shown in Figure 11a, the absolute value of thermopower is proportional to the energy differential in the density of the state:

$$|S|\;\propto {\left[\frac{\partial DOS(E)}{\partial E}\right]}_{E=E_{F}}$$

As the thickness of the 2DES decreases below the de Broglie Wavelength, the conduction band (CB) will split, which results in an enhancement of thermopower. In 2007, Ohta et al. fabricated the SrTi_0.8_Nb_0.2_O_3_|SrTiO_3_ superlattice structure and confirmed the 2DES enhancement effect in thermopower by decreasing the thickness of the quantum wells (Figure 11b) [73]. A *ZT* value of ~2.4 was achieved in the 2DES (Figure 11c). 

**Figure 12:**
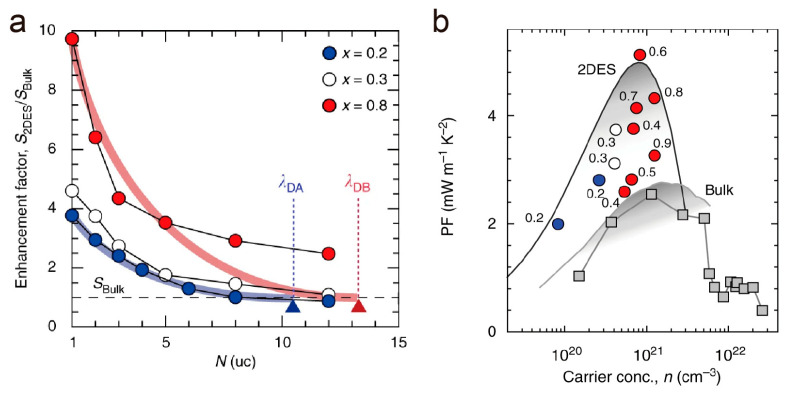
Longer de Broglie wavelength creates a larger enhancement in thermoelectric performance. (**a**) Enhancement factors in thermopower (*S*_2DES_/*S*_Bulk_) for three sets of [*N* uc SrTi_1−*x*_Nb*_x_*O_3_|11 uc SrTiO_3_]_10_ 2DESs. For *x* = 0.2 and 0.3 2DESs, the highest *S*_2DES_/*S*_Bulk_ values are obtained at *N* = 1, which are 4 and 5, respectively, while that of *x* = 0.8 can reach 10. (**b**) Double enhancement of power factor (PF) is seen when *x* = 0.6 (5.1 mW m^−1^ K^−2^ at *n*~8 × 10^20^ cm^−3^). Reproduced with permission [75].

In order to achieve a stronger enhancement effect, Dresselhaus et al. proposed the concept of using materials with longer de Broglie wavelengths in quantum well constructions [74]. Based on this concept, Zhang et al. utilized the tunable de Broglie wavelength in the SrTiO_3_–SrNbO_3_ full-range solid solution when constructing superlattices [69]. With a longer de Broglie wavelength, the superlattice structures achieved superior overall thermoelectric performances over the bulk counterparts [75]. As shown in Figure 12a, the 2DES exerts a much higher enhancement factor (*S*_2DES_/*S*_Bulk_) in the thermopower, where the 2DES with a longer de Broglie wavelength reached 10, which is ~2 times larger than the 2DESs, which has a shorter de Broglie wavelength. Meanwhile, a longer de Broglie wavelength better maintains the better electron-transport property. As a result, the highest power factor in the superlattices reached ~5 mW m^−1^ K^−2^, thereby reaching 200% of the bulk counterparts (Figure 12b).

More recently, the strategy of high-entropy alloying has been applied to increase the thermoelectric performance of SrTiO_3_-based materials, where multiple metal ions are introduced to the A-site or B-site of the SrTiO_3_ lattice to induce more anharmonicity and multiphonon scattering, while the lattice thermal conductivity can be significantly suppressed. Banerjee et al. mixed five transition metal elements at the B-site in the SrTiO_3_, synthesizing Sr(Ti_0.2_Fe_0.2_Mo_0.2_Nb_0.2_Cr_0.2_)O_3_ high-entropy perovskite ceramics. The thermal conductivity can be reduced to 0.7 W m^−1^ K^−1^ at 1100 K [76]. Zheng et al. fabricated the A-site high-entropy SrTiO_3_-based thermoelectric ceramics [77]. Ca, Sr, Ba, Pb, and La were introduced to induce a large lattice distortion and a huge mass fluctuation effect, which resulted in a minimum thermal conductivity of 1.17 W m^−1^ K^−1^ at 923 K. Using newer concepts combined with traditional oxide-based materials, a chance remains to further increase their thermoelectric performance. 

## 4. Layered Cobalt Oxides (*A_x_*CoO_2_ (*A_x_* = Na_0.75_, Ca_0.33_, Sr_0.33_, Ba_0.33_))

**Figure 13:**
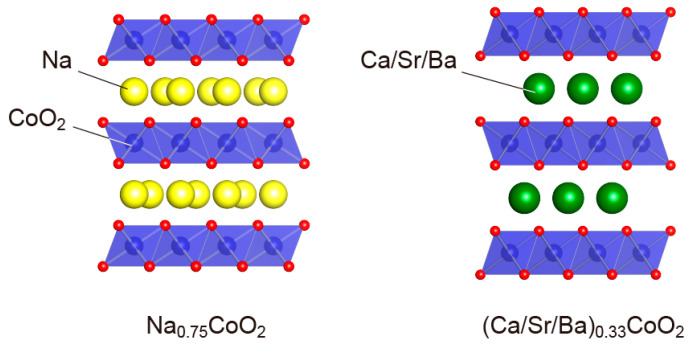
Schematic illustration of the layered cobaltite crystal structure (*A_x_*CoO_2_ (*A_x_* = Na_0.75_, Ca_0.33_, Sr_0.33_, Ba_0.33_)).

Layered cobalt oxides (*A_x_*CoO_2_) exert a sandwich structure composed of CoO_2_ and A-site cations by stacking layer by layer (Figure 13). The conduction path is dependent on the CoO_2_ layer, which displays a large Seebeck coefficient due to the low-spin state of Co^3+^ [78]. It has been theoretically and experimentally proven that the spin entropy flow generated from the electronic conduction between Co^3+^ and Co^4+^ majorly contributes to the enhancement of the thermoelectric potential of *A_x_*CoO_2_-based oxide materials [78,79,80,81]. At high temperatures, the Seebeck coefficient (*S*) can be calculated based on the Koshibae equation:

$$S\;(T\to \infty )\;=-\frac{k_{B}}{e}\;\ln\left[\frac{g({Co}^{4+})}{g({Co}^{3+})}\;\frac{n_{{Co}^{4+}}}{n_{{Co}^{3+}}}\right]$$

where *k_B_* is the Boltzmann constant, *e* is the electronic unit charge, *g* is the different possible ways in which electrons can be arranged in the orbitals of Co^3+^ and Co^4+^ ions, and *n* is the concentration of a given Co species. Additionally, *g* is the product of the spin degeneracy (*g_s_*) and orbital (*g_o_*) degeneracy: *g* = *g_s_*…*g_o_*, whereby *g_s_* = 2*ζ* + 1, where *ζ* is the ions’ total spin number and *g*_o_ is the number of valid permutations for distributing the electrons across its orbitals. Using electronic conduction through hopping in Na*_x_*CoO_2_, a spin entropy flow occurs in the opposite direction [82]. Therefore, the contribution of the spin entropy to the thermoelectric potential in the cobalt oxide group is related to the concentration of the Co^4+^ and Co^3+^ ions, as well as the degree of the spin degeneracy of Co ions. Since a decrease in the Co^4+^ concentration will lead to an increase in spin entropy; thus, the manipulation of the Co^4+^ ion concentration through excessive metal doping has emerged as an effective approach to modulating the spin entropy contribution. Using the ion exchange process, the A-site cations can be tuned from Na^2+^ to Ca^2+^, Sr^2+^, and Ba^2+^ and accompanied by a topotactic phase transition. 

In 1997, Terasaki et al. discovered that Na_0.5_CoO_2_ crystal was a good candidate for thermoelectric application [35]. Compared to the traditional alloy-type materials Bi_2_Te_3_, it was found that their power factors are in the same order of magnitude. Meanwhile, the conductivity of Na*_x_*CoO_2_ is nearly four times that of Bi_2_Te_3_ [35,83]. In terms of thermal conductivity, the interface between layers affects heat transfer and contributes to the reduction in lattice thermal conductivity. Therefore, the large Seebeck coefficient and high electrical conductivity also indicate that the layered Na_0.5_CoO_2_ crystal is a kind of oxide-based thermoelectric material with research significance and prospect. The discovery has also drawn the attention of researchers to various metallic layered cobalt oxide-based thermoelectric materials.

**Figure 14:**
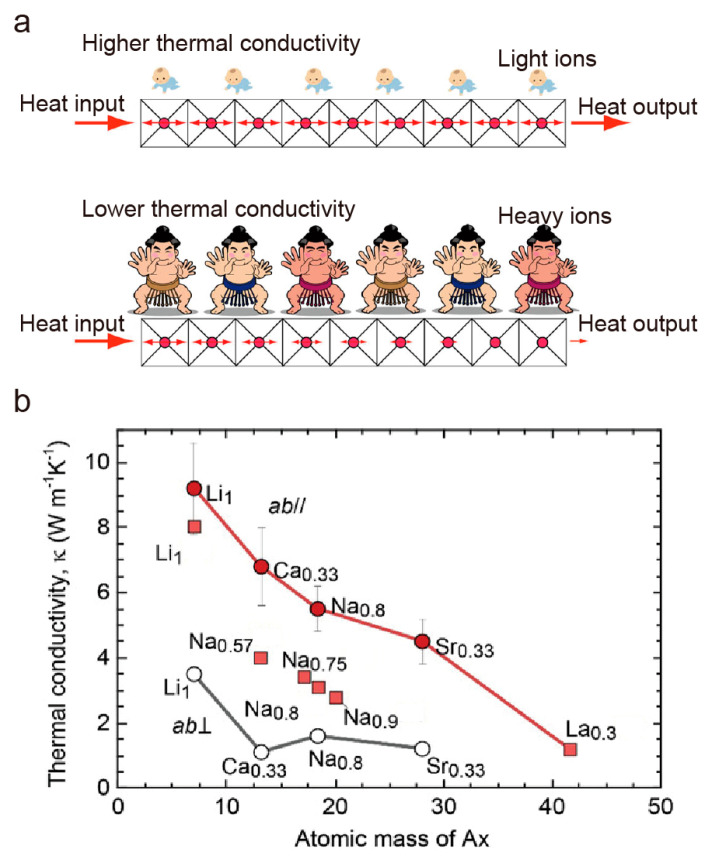
Reduction in thermal conductivity through heavy ion substitution at the *A_x_*CoO_2_ A-site. (**a**) Schematic phonon propagation of *A_x_*CoO_2_. Heavy ions (sumo wrestler) display a stronger thermal conductivity suppression effect than light ions (baby). (**b**) Anisotropic thermal conductivities of *A_x_*CoO_2_. As the mass of A-site ions increases, the in-plane thermal conductivity (*κ*_||_) and cross-plane thermal conductivity (*κ*_⊥_) decrease in tendency. Reproduced with permission [84].

However, constrained by the high thermal conductivity, the overall thermoelectric performance, *ZT* value, of Na_0.5_CoO_2_ is still far from the commercial level. In 2020, Cho et al. proposed that thermal conductivity can be suppressed significantly by substituting A-site ions in *A_x_*CoO_2_ from light ions to heavy ions [84]. Using heavier A-site ions will create a mismatch in phonon vibration modes between CoO_2_ and the A-site cation layers, which will reduce the phonon mean free path and phonon group velocity, and therefore, the thermal conductivity (Figure 14a). They fabricated *A_x_*CoO_2_ (*A_x_* = Li_1_, Na_0.75_, Ca_0.33_, Sr_0.33_, La_0.3_) epitaxial films using the PLD method followed by the reactive solid phase epitaxy (R-SPE) method and ion exchange process. The thermal conductivity tendency was good after following the hypothesis along the in-plane direction, suggesting that the thermoelectric performance of *A_x_*CoO_2_-based materials can be enhanced through an elaborate modification of the A-site ions. 

**Figure 15:**
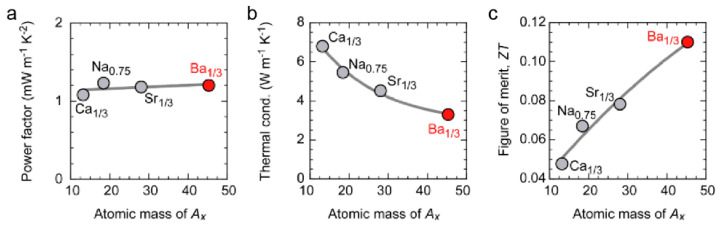
Thermoelectric properties of *A_x_*CoO_2_ epitaxial films at room temperature. (**a**) Power factor, (**b**) thermal conductivity, and (**c**) *ZT* values. The thermal conductivity decreases with A-site ion mass, while the power factor stays constant. The *ZT* value of Ba_0.33_CoO_2_ epitaxial film is ~0.11. Reproduced with permission [40].

Following this strategy, Takashima et al. checked the thermoelectric properties of *A_x_*CoO_2_ (*A_x_* = Na_0.75_, Ca_0.33_, Sr_0.33_, Ba_0.33_) epitaxial films along the in-plane direction [40]. As shown in Figure 15, the power factor stays almost unchanged as the A-site ion mass increases, while the thermal conductivity is effectively suppressed, which suggests that the A-site ion exchange will not affect the electrical conduction path along the CoO_2_ layer and only reduce their thermal transport properties. It also needs to be emphasized that *A_x_*CoO_2_ shows an A-site ion arrangement evolution with temperature. For example, Ca_0.33_CoO_2_ exerts a phase transition from a $\sqrt{3}a\times \sqrt{3}a$ hexagonal superstructure to a $2a\times \sqrt{3}a$ orthorhombic superstructure as the temperature increases above 400 °C [85]. However, in the Ba_0.33_CoO_2_ lattice, a mixture of orthorhombic and hexagonal superstructures can be maintained within a wide temperature range. This lattice disorder contributes significantly to phonon scattering and thermal conductivity reduction. A peak *ZT* value of ~ 0.11 was realized in the Ba_0.33_CoO_2_ epitaxial film at room temperature, which is a record high among the reported “reliable” *ZT* values in conducting oxides. 

**Figure 16:**
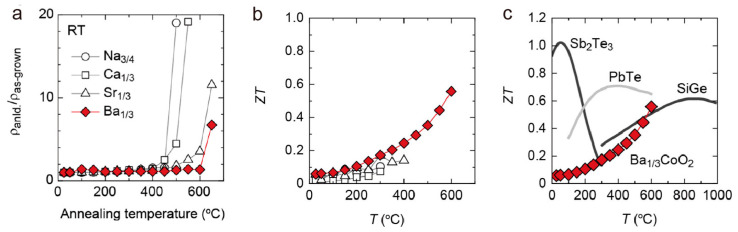
Thermal stability and thermoelectric properties of *A_x_*CoO_2_ (*A_x_* = Na_3/4_, Ca_1/3_, Sr_1/3_, and Ba_1/3_) epitaxial films. (**a**) Resistivity was measured at room temperature after annealing at high temperatures for 0.5 h in air. (**b**) Temperature-dependent ZT values compared among the four *A_x_*CoO_2_ (*A* = Na_3/4_, Ca_1/3_, Sr_1/3_, and Ba_1/3_) films and (**c**) against commercially available p-type thermoelectric materials. Reproduced with permission [41].

As indicated in the report by Takashima et al., Ba_0.33_CoO_2_ is a promising thermoelectric material with superior performance to other counterparts. Later, Zhang et al. tested the high-temperature thermoelectric properties of *A_x_*CoO_2_ (*A_x_* = Na_0.75_, Ca_0.33_, Sr_0.33_, Ba_0.33_) epitaxial films to confirm the application of Ba_0.33_CoO_2_ at elevated temperature environments [41]. They found a relatively high thermal stability of Ba_0.33_CoO_2_ up to 650 °C in air compared to other counterparts, by checking the room temperature resistivity after heat treatment in air at elevated temperatures (Figure 16a). High thermal stability makes it possible to measure thermoelectric properties of Ba_0.33_CoO_2_ up to 600 °C, with a *ZT* value of 0.55 at 600 °C (Figure 16b), comparable to those of PbTe and p-type SiGe (Figure 16c), which indicates Ba_1/3_CoO_2_ as a good candidate for high-temperature thermoelectric applications.

## 5. Summary and Perspective

The recent advancements in oxide-based thermoelectric materials, with a focus on ZnO, SrTiO_3_, and layered cobalt oxides, are reviewed, as they have garnered increasing attention due to their advantages over the chalcogenide family. The combination of high thermal/chemical stabilities and abundant elements on Earth makes thermoelectric oxides a highly promising candidate for numerous applications in waste heat recycling at elevated temperatures. Until now, these materials have witnessed revolutionary breakthroughs in performance enhancement through conventional doping and nanostructuring techniques. Additionally, the concept of low-dimensionalization has emerged as a new approach to further improve efficiency, especially in the context of device miniaturization and flexibility. With the implementation of novel strategies aimed at enhancing the performance of thermoelectric oxides, such as augmenting the power factor through low-dimensionalization and reducing thermal conductivity via high-entropy alloying, oxide-based thermoelectric materials are poised to undergo significant advancements in the foreseeable future.
